# Closing Editorial

**DOI:** 10.3390/polym16243594

**Published:** 2024-12-22

**Authors:** Raffaella Striani

**Affiliations:** Department of Engineering for Innovation, University of Salento, Via Monteroni, 73100 Lecce, Italy; raffaella.striani@unisalento.it

It is a real honor for me to be the Guest Editor of this Special Issue and to continue the project with the second edition “Sustainable Biopolymer-Based Composites: Processing, Characterization and Application II”.

The great success it has had, and continues to have, is proof that the subject of sustainable biopolymer-based composites is alive and in continuous growth. With a collection of 32 papers, every single author has contributed to the first highly successful volume.

The topic has found ample space in the world of scientific research and has had the opportunity to be analyzed in various sectors. The wide range of applications in which the study of biopolymer-based composites has be applied, from processing to characterization, is the added value of this Special Issue. Packaging, waste valorization, sports, green transition, skin pigmentation disorders, agriculture, recyclable resins, dental prostheses, microfluidic devices, are among the main applications addressed in the reviews and scientific articles collected in this volume, which have taken biopolymers as object of study, including but not limited to: chitosan [1], wood waste [2], lignin [3,4,5,6], collagen [7,8], polylactic acid [9,10,11], bioplastics [12,13], cellulose [14,15,16], starch [17,18,19,20,21], vegetable fibers [22], animal fibers [23,24], natural-based hydrogels [25].

The way is still open. The issue of sustainability is becoming increasingly topical. Therefore, I invite all scientists to contribute with their passion and scientific commitment to share their research in the field of biopolymer-based composites in the second volume Sustainable Biopolymer-Based Composites: Processing, Characterization and Application II.
